# Supplementary material for: Nature of the Unconventional Heavy-Fermion Kondo State in Monolayer CeSiI
Source: Nano Lett. 2024 Feb 23;24(14):4272–8. doi: 10.1021/acs.nanolett.4c00619 (PMC11010227; doi:10.1021/acs.nanolett.4c00619)
Supplement: Supplementary file 1 — nl4c00619_si_001.pdf [file nl4c00619_si_001.pdf]

Supplemental Information: Nature of the unconventional heavy fermion Kondo state in monolayer CeSiI

Adolfo O. Fumega[1,*] and Jose L. Lado[1,†]
[1]*Department of Applied Physics, Aalto University, 02150 Espoo, Finland*

DFT BAND STRUCTURE IN THE ABSENCE OF SPIN-ORBIT COUPLING

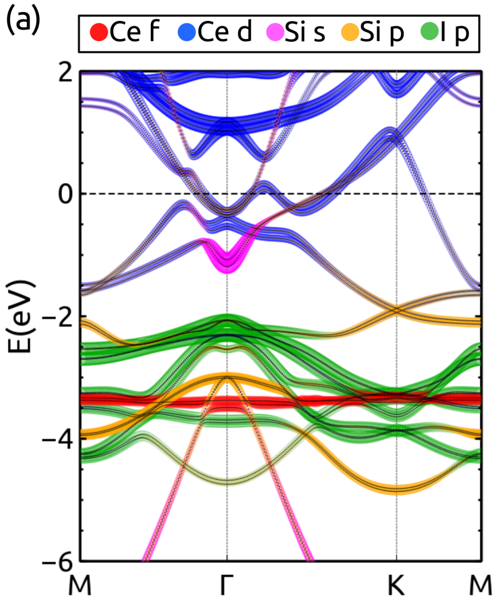

FIG. S1. (a) DFT orbital-resolved band structure of CeSiI in the absence of spin-orbit coupling

The electronic band structure of monolayer CeSiI in the absence of spin-orbit coupling is shown in Fig. S1.

STRAIN DEPENDENCE OF THE KONDO LATTICE PARAMETERS

The tunability to go from the unconventional heavy fermion to the conventional one could be studied as a function of strain. This is controlled by the J_1/J_K ratio. In order to illustrate that, we have computed the evolution of this ratio as a function of strain (Fig. S2c). The Kondo coupling is enhanced for tensile strain (Fig. S2a). This effect can be understood considering that tensile strain induces a reduction of the conduction bands' bandwidth, thus causing an increase in the density of states at the Fermi level and consequently an increase of the Kondo coupling in absolute value. The magnetic exchange shows a small oscillatory behavior as a function of strain presumably due to the RKKY interaction that dominates it (Fig. S2b). Considering the evolution of the $|J_1/J_K|$ ratio (Fig. S2c), we can observe that the ratio decreases for tensile strain, thus driving the system from the unconventional heavy fermion phase to the conventional one.

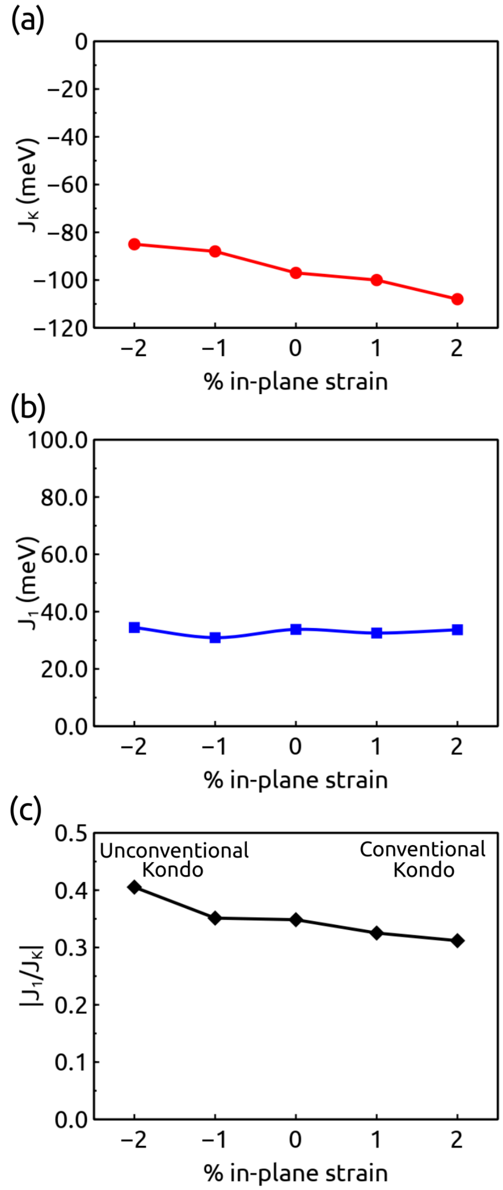

FIG. S2. Evolution of J_K (a), J_1 (b), and the J_1/J_K ratio (c) as a function of in-plane strain.

A similar trend for the evolution of the heavy-fermion behavior as a function of pressure has been reported in other Ce-based Kondo insulators such as CeNiSn [1], CeRhSb[2] or $Ce_3Bi_4Pt_3$ [3]. The Kondo gap closes upon

applying pressure, i.e. decreasing the Kondo hybridization. This occurs despite the general consideration that reducing unit cell volume should increase the Kondo hybridization. In a more complex and realistic multiorbital scenario, inverse pressure (or tensile strain) can promote a heavy-fermion regime. For the case of monolayer CeSiI, further experiments will be required to elucidate this predicted trend.

* adolfo.oterofumega@aalto.fi
† jose.lado@aalto.fi

[1] M. Kurisu, T. Takabatake, and H. Fujiwara, "Gap suppression in cenisn under hydrostatic pressure," Solid State Communications **68**, 595–597 (1988).
[2] Yoshiya Uwatoko, Takayuki Ishii, Gendo Oomi, Hiroki Takahasi, Nobuo Mōri, J. D. Thompson, J. L. Shero, D. Madru, and Z. Fisk, "Pressure collapse of kondo gap in kondo compound cerhsb," Journal of the Physical Society of Japan **65**, 27–29 (1996).
[3] Daniel J. Campbell, Zachary E. Brubaker, Connor Roncaioli, Prathum Saraf, Yuming Xiao, Paul Chow, Curtis Kenney-Benson, Dmitry Popov, Rena J. Zieve, Jason R. Jeffries, and Johnpierre Paglione, "Pressure-driven valence increase and metallization in the kondo insulator $ce_3bi_4pt_3$," Phys. Rev. B **100**, 235133 (2019).
